# Present and Future of Dengue Fever in Nepal: Mapping Climatic Suitability by Ecological Niche Model

**DOI:** 10.3390/ijerph15020187

**Published:** 2018-01-23

**Authors:** Bipin Kumar Acharya, Chunxiang Cao, Min Xu, Laxman Khanal, Shahid Naeem, Shreejana Pandit

**Affiliations:** 1 State Key Laboratory of Remote Sensing Science, Institute of Remote Sensing and Digital Earth, Chinese Academy of Sciences, Beijing 100094, China; bipin@radi.ac.cn (B.K.A.); xumin@radi.ac.cn (M.X.); shahid.613@gmail.com (S.N.); 2 University of Chinese Academy of Sciences, Beijing 100049, China; laxman@mail.kiz.ac.cn; 3 Kunming Institute of Zoology, Chinese Academy of Sciences, Kunming 650223, China; 4 Central Department of Zoology, Institute of Science and Technology, Tribhuvan University, Kathmandu 44613, Nepal; 5 Kanti Children’s Hospital Maharajgunj, Kathmandu 44616, Nepal; shreejanapandit@hotmail.com

**Keywords:** dengue mapping, climate change, Nepal, MaxEnt

## Abstract

Both the number of cases of dengue fever and the areas of outbreaks within Nepal have increased significantly in recent years. Further expansion and range shift is expected in the future due to global climate change and other associated factors. However, due to limited spatially-explicit research in Nepal, there is poor understanding about the present spatial distribution patterns of dengue risk areas and the potential range shift due to future climate change. In this context, it is crucial to assess and map dengue fever risk areas in Nepal. Here, we used reported dengue cases and a set of bioclimatic variables on the MaxEnt ecological niche modeling approach to model the climatic niche and map present and future (2050s and 2070s) climatically suitable areas under different representative concentration pathways (RCP2.6, RCP6.0 and RCP8.5). Simulation-based estimates suggest that climatically suitable areas for dengue fever are presently distributed throughout the lowland Tarai from east to west and in river valleys at lower elevations. Under the different climate change scenarios, these areas will be slightly shifted towards higher elevation with varied magnitude and spatial patterns. Population exposed to climatically suitable areas of dengue fever in Nepal is anticipated to further increase in both 2050s and 2070s on all the assumed emission scenarios. These findings could be instrumental to plan and execute the strategic interventions for controlling dengue fever in Nepal.

## 1. Introduction

Dengue fever is a mosquito-borne viral disease transmitted by the female *Aedes* mosquito, especially the *Aedes aegypti*. In recent years, dengue has been a serious public health concern of tropical and subtropical countries across the world. It has been estimated that around 390 million infections occur annually and about 3.9 billion people are under the direct risk of the disease [1]. The burden of disease has increased 30-fold over the past 50 years [2] and the number of dengue-endemic countries increased from 9–125 in the last 40 years [3]. Further geographic expansion of the disease is expected in the future due to climate change, urbanization, and migration [4].

Dengue is a climate-sensitive disease. Temperature and precipitation are the most important climate factors in the occurrence and transmission of dengue fever [5]. These climate factors affect mosquito population (survival and development), virus propagation (replication) and vector-host interaction (biting rate) [6]. Increased temperature alters vectorial capacity by reducing the duration of mosquito development rate, vector competence and extrinsic incubation period [7,8,9]. The right temperature and level of precipitation are important to provide the necessary environment for the development of the mosquito vector, but thermal extremes and heavy rainfall are negatively associated with the disease [10]. Therefore, global geographic distribution of the dengue has been determined by latitude as a proxy to temperature, whereas, on a local scale, its distribution has been limited by altitude as a proxy for thermal variation [11].

The climate change issue remained one of the controversial focus points in the last few decades. Recently, scientists have largely agreed on attributing global warming to an increasing concentration of greenhouse gases in the atmosphere. According to the recent International Panel on Climate Change (IPCC) report, the average global temperature has increased by 0.85 °C from 1880–2012 and is likely to increase further by a minimum of 0.3–1.7 °C (representative concentration pathway (RCP) 2.6) and a maximum of 2.6 to 4.8 °C (RCP8.5) by the end of the century relative to the 1986–2005 temperature [12]. Due to the close link with climate factors, many experts hypothesize that the dengue incidence will increase in the future by expanding geographic range [13,14,15,16,17]. Increases in the global temperature can cause latitudinal and altitudinal shifts in the ecological niche of vector-borne infectious diseases [14]. Improvement in our understanding of how the changing climate may contribute to the geographic expansion of mosquitoes and the disease into new areas is very crucial to support the public health authorities for effective surveillance and control strategies.

Several efforts have been made earlier to map distribution of the dengue fever in global [1], regional [18], national, and subnational scales [19]. Geographic Information System (GIS) and Remote Sensing (RS) technology and more recently, the ecological niche based species distribution modeling (SDM) approach have made remarkable breakthroughs in this field, especially in the availability of geospatial data, computational capacity and the strength of visualization. SDM offers several advantages over the traditional mapping techniques (data driven descriptive choropleth mapping and analytical spatial interpolation methods) [20]. Unlike the traditional mapping technique, SDM can produce a more accurate and robust map even with an incomplete and noisy dataset [19,20]. This is the most important advantage of the SDM in disease mapping as collection of disease data is difficult due to underreporting, misdiagnosis and ethical issues of personal identity. SDM establish relationships between known locations of disease with potential environmental covariates then predict distribution in a space and time. Using these methods, several previous studies [1,19,21] were able to widen our understanding not only on the present distribution of dengue fever but also on possible impact of climate change in the future [21]. Such studies successfully identified the risk factors associated with them. MaxEnt, Boosted Regression Tree (BRT), Random Forest are some of the widely used machine learning SDM algorithms. Among others, MaxEnt, which requires presence-only data (instead of presence/absence) is widely used among the mapping methods due to robust predictive performance compared to other contemporary methods [22].

Dengue fever cases have been reported every year from southern lowland Tarai in Nepal since its emergence in 2004. In 2010 and 2013, Nepal experienced two large outbreaks with 917 confirmed cases and five deaths, and 642 confirmed cases respectively [23]. These statistics are believed to be underreported and the prevalence of dengue is considered much higher [24]. Lowland Tarai especially Chitwan and Jhapa district were severely affected districts in those outbreaks. Recently the disease has been reported from higher elevated mid hills. Nepal is one of the vulnerable countries from the global climate change perspectives. The region has warmed up by 1.5 °C in the last 25 years with much higher rates compared to the global average and is expected to be warmer and wetter in the future due to climate change [25]. In this context dengue is likely to be shifted toward higher elevation [26] in response to such climate change. The recent study documented the presence of *A. aegypti* up to 2000 m above sea level (asl) [27] and all the serotypes of dengue virus circulate among host, vector and environment in the country [28,29]. The dengue problem can be anticipated to get much worse in the future. However, climate-based spatial risk assessment is lacking in Nepal, both for the present and for the future. To support the surveillance and dengue control strategies, it is imperative to have such a mapping assessment. To overcome this lack, we mapped present and future climatically suitable areas of dengue fever in Nepal based on reported dengue cases collected from different sources and a set of bioclimatic variables using the ecological niche model. Based on the model, we estimated the proportion of people/area at risk of the disease.

## 2. Materials and Methods

### 2.1. Study Area

**Figure 1:**
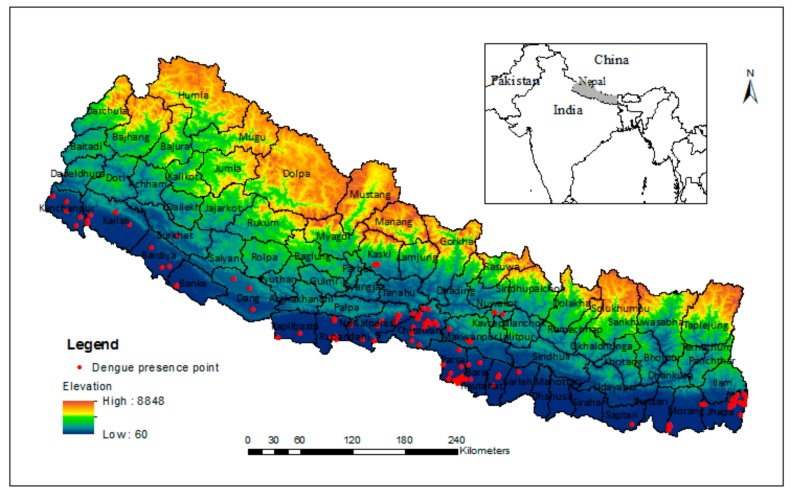
Study area showing elevation gradient and location of reported dengue cases in Nepal.

Nepal is approximately located between a latitude of 26° to 30° North and a longditude of 80° to 88° East on the southern slope of the central Himalayas (Figure 1). The total area of the country is 147,181 km^2^. There is a remarkable difference in elevation ranging from 60 m above sea level (asl) in the southern lowland to 8848 m asl in Himalayas in the north. Based on the altitudinal gradient, Nepal is divided into Tarai, Chure, Mahabharat and Himalaya as broad physiographic zones from south to north. The climate of Nepal is broadly subtropical monsoon characterized by large seasonal variations in temperature, rainfall and humidity. At the local scale, the north–south elevation gradient controls the climate of Nepal. The southern part is hot most of the year, whereas high mountains in the north remain frozen all year round. Nepal receives an average annual rainfall of 1600 mm, 80% of which occurs during the four wet months (June–September) of the monsoon period following the hot summer season. The winter season in Nepal is relatively cold and dry and receives only about 20% of the annual rainfall from the westerly direction. The spatial distribution of rainfall also varies due to sharp topographical variations. The southern windward side receives more rainfall compared to the northern leeward side [30].

### 2.2. Dengue Fever Data

The presence location of the study subject is the first essential dataset for ecological niche modelling. In the field of disease ecology some studies use entomological data, i.e., presence location of the vector; whereas, other studies use an epidemiological dataset, i.e., presence location of reported disease cases. Epidemiological data is considered more representative of the actual suitability given their inherent linkage to an infected individual [19,31,32]. Therefore, we used an epidemiological dataset in this study. We collected dengue fever data primarily from three different sources, because, the complete coverage of dengue data was not available from a single source such as government records. The majority of them were retrieved from the line listing file; a patient address log book at Epidemiology and Disease Control Division (EDCD), Department of Health Services, Government of Nepal. Similarly, we also collected dengue presence locations from national newspaper portals. The third source of dengue data was the health map geoportal (http://www.healthmap.org/en/). All the sources of data were confirmed dengue cases reported to the authority based on laboratory tests. The dengue data from the first two sources were address level data without geolocation attached, so, we geocoded them using Google API (Application Programming Interfaces) in R (Supplementary Note S1). Finally, 178 dengue presence geolocations were filtered based on one square kilometer grids and duplications were removed, retaining 124 dengue presence locations (Table S1) for the ecological niche modelling (ENM).

### 2.3. Predictor Variables

We retrieved 19 bioclimatic variables (version 1.4) in a 30 arc second spatial resolution (Table 1) from the WorldClim global climate database (http://www.worldclim.org) [33] to represent the present bioclimatic conditions. This dataset was generated by interpolation using a thin-plate smoothing spline of observed climate from worldwide weather stations for the period of 1950–2000, with latitude, longitude and elevation as independent variables [33]. These data are biologically meaningful variables that capture annual ranges, seasonality, and limiting factors useful for niche modeling (such as monthly and quarterly temperature and precipitation extremes) [33]. Elevation was not explicitly used in model construction because it has already been used as a covariate in the Worldclim data production [34].

To estimate the impacts of plausible future climate conditions [12], we selected the Community Climate System Model (CCSM) representing three future greenhouse gases concentration trajectories, also known as representative concentration pathways (RCP2.6, RCP6.0 and RCP8.5), for two different time periods (2050s and 2070s) as adopted by the International Panel on Climate Change (IPCC) in its fifth Assessment Report (AR5). The selected RCPs represent four possible greenhouse gas emission scenarios ranging from low (RCP2.6) to high (RCP8.5) corresponding to increases in global radiative forcing values in the year 2100 relative to preindustrial values (2.6, 4.5, 6.0 and 8.5 w/m^2^), respectively. Future climate projection data were also downloaded from the Worldclim geoportal (http://www.worldclim.org/).

### 2.4. Modeling and Validation

We used the Maximum entropy modeling (MaxEnt) [35] approach using the standalone MaxEnt software. MaxEnt is a machine learning program that uses presence-only data to predict distributions based on the principle of maximum entropy. The basic principle of the MaxEnt model is to estimate the potential distribution of a species by determining the distribution of the maximum entropy (i.e., closest to uniform), with constraints imposed by the observed spatial distributions of the species and the environmental conditions [35].

We used 124 dengue presence locations and a set of bioclimatic raster layers as an input to run the model. As bioclimatic data in Worldclim are derived from a common set of temperature and precipitation, the variables can exhibit multicollinearity [33]. The relative contribution of each predictive variable, which is normally interpreted using the variable contribution table and jackknife test, is affected by high correlation among the predictor variables [19]. As a result, ecologically more causal variables can be excluded from models if other correlated variables explained the variation in the response variable better in statistical terms [36]. We therefore computed the Pearson correlation coefficient to remove highly correlated variables using the correlation threshold value of >|0.7| (Table S2). Further, we assessed the statistical contribution of each of the variables by computing univariate model and observing the area under the curve (AUC) of the receiver operating characteristic (ROC) statistics (Figure S1). We also considered the biological importance of the variables in the variables selection approach. Overall, our variables selection approach is similar to Ren et al. [37] and TuanMu et al. [38]. Finally, we selected three variables: the mean temperature of the wettest quarter (bio8), the mean temperature of the coldest quarter (bio11) and the precipitation seasonality (bio15) to run the final model. For the national level study, three to four bioclimatic variables were found to be the best in describing the ecological niche of dengue fever [19]. Finally, the three selected variables were converted to ASCI (American Standard Code for Information Interchange) format and were used as the predictor variables to develop the final MaxEnt model. To simulate the climatically suitable areas under possible future climatic conditions, the same variables (bio8, bio11, and bio15) were set as the input in the projection layers directory.

To evaluate model performance, we divided data randomly into training (75%) and validation datasets (25%). Then, we used the threshold independent AUC to estimate the performance of the model. AUC measures the predictive performance of the model by comparing the model’s predictive ability to the random prediction. The AUC value ranges from 0 to 1 where 0.5 indicates random prediction and higher values correspond to a better model [35]. In order to take account of uncertainty introduced by training and validation set splits, 30 models were produced by 30 replicate runs using the cross validation approach [37]. Maximum, minimum and standard deviation of the replication were used to evaluate the possible biases generated due to the arbitrary data split. All the data were used to make the final predictions. The nonlinear functional relationship between dengue fever and climate factors was evaluated through inspection of the response curves. Response curves show how the y-axis (response as relative index of occurrence) relates to changes along the x-axis (a single predictor when all others are held constant and corrected for). The relative importance of each environmental variable in the model was evaluated by the Jackknife test and variable contribution table. The logistic output was used in MaxEnt, which generates a continuous map with an estimated probability of presence between 0 and 1.

Finally, a climate suitability map was produced with values ranging from 0 (completely unsuitable) to 1 (the most favorable conditions) that were reclassified into unsuitable (<0.297), moderately suitable (0.297–0.45) and highly suitable (>0.45) based on 10th percentile presence threshold (0.297) [39] and the minimum training presence area (0.45) over the 30 model iterations. We did this for both the present and future climate conditions under different emission scenarios and both the time periods. To evaluate the potential altitudinal shift of the climatic niche, we overlaid both the present and future climate suitability maps to the Shuttle Radar Topographic Mission (SRTM) digital elevation model (DEM) (http://www.cgiar-csi.org/data/srtm-90m-digital-elevation-database-v4-1) and extracted elevation values of each of the climate suitable pixels. Then, we plotted these values and computed the mean, maximum and minimum to understand altitudinal limit of the climatic niche of dengue fever. By this, we assessed distribution of climatically suitable pixel of dengue fever along elevation gradient including upper and lower altitudinal limit. We used a grid-based population dataset available at one kilometer pixel resolution for the year 2010 collected on the worldpop geoportal (http://www.worldpop.org.uk/) to estimate the present exposed human population in the dengue climatic niche. Future potential human population exposure to dengue fever for the 2050s and 2070s was estimated by linear interpolation based in the annual population growth rate of 2011 national census [40] of Nepal.

## 3. Results

**Figure 2:**
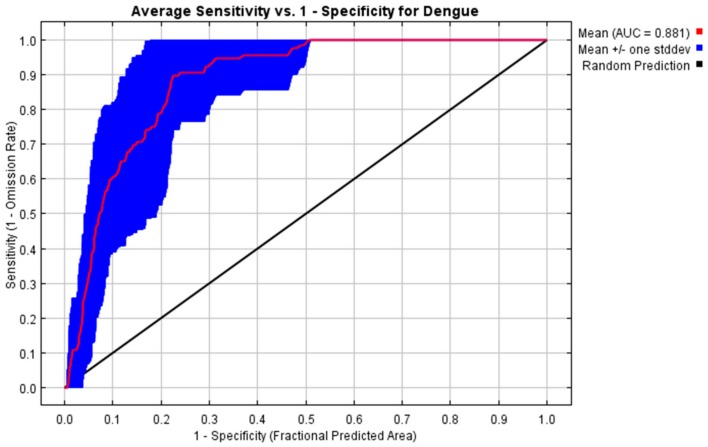
Average area under curve (AUC) for 30 replicates MaxEnt run. The red line is the average value and the blue bar represents plus and minus one standard deviation.

**Figure 3:**
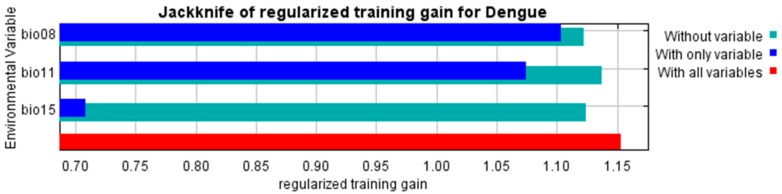
Variable importance by Jackknife test. The blue, aqua and red bars represent the results of the model created with each individual, all remaining variables and all the variables respectively.

The MaxEnt model developed in this study performed well with a mean training AUC of 0.8815 ± 0.0419 and a mean test AUC of 0.8815 ± 0.057 of 30-fold cross validation indicating the robust prediction of distribution of dengue suitable areas by the selected variables (Figure 2). The relative contributions of each environmental variable to the MaxEnt model are shown in Figure 3. The bio8 accounted for 61.16% of the relative contribution to the model, followed by bio11 (29.26%) and bio15 (9.56%). The MaxEnt model’s Jackknife test of variable importance showed that bio8 (mean temperature of the wettest quarter) was the variable with the highest gain when used in isolation. It indicated a strong contribution of bio8 to the model development that has the most useful information among the variables. Further, omission of bio8 decreased the gain of model. Such a result indicates that it holds the most information for dengue distribution among the variables used for model development (Figure 2).

According to the response curve plots (Figure S2), bio8 in the range of 25–28 °C, bio11 between 13.5–16.5 °C and bio15 between 105–135 °C were ideal to define moderately suitable areas for dengue fever. Similarly, to be a highly suitable climatic niche, the critical range of these variables should be in the range of 28–30 °C, 16.5–17.5 °C and 135–145 °C, respectively.

**Figure 4:**
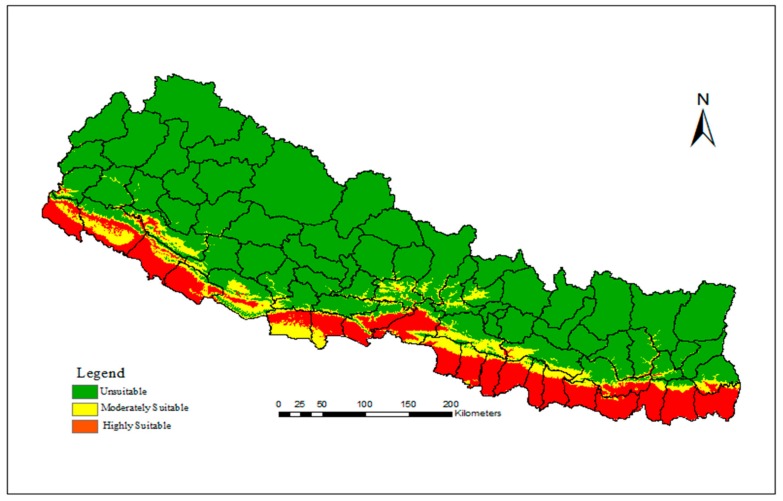
Spatial distribution of present day climatically suitable areas of dengue fever in Nepal obtained from MaxEnt modelling approach.

Figure 4 shows the spatial distribution of present day climatically suitable areas of dengue fever in Nepal. It is distributed throughout the southern lowland Tarai; from east to the west and also in some less elevated river valleys in the Hill and Mountain regions. The distribution of suitable areas is continuous from east to the west except in the mid-west around the Dang district (see Figure 4). Chure, which lies immediately north of lowland Tarai, behaves as the northern limit of dengue fever with the exceptions of low elevated river valleys in the hilly region. According to our model, about one-fourth (24.43%) area of the country is presently climatically suitable for dengue fever (9.33% moderately suitable, and 15.09% highly suitable). The moderately suitable areas were generally observed in the northern margin of highly suitable areas, however, they were intermixed in many parts of western Tarai. The climatically suitable area was observed to decrease with increasing elevation from south to the north (Figure S3). The highest concentration of climatically suitable area pixels was observed in less than 300 m asl and the lowest in 800–900 m asl.

**Figure 5:**
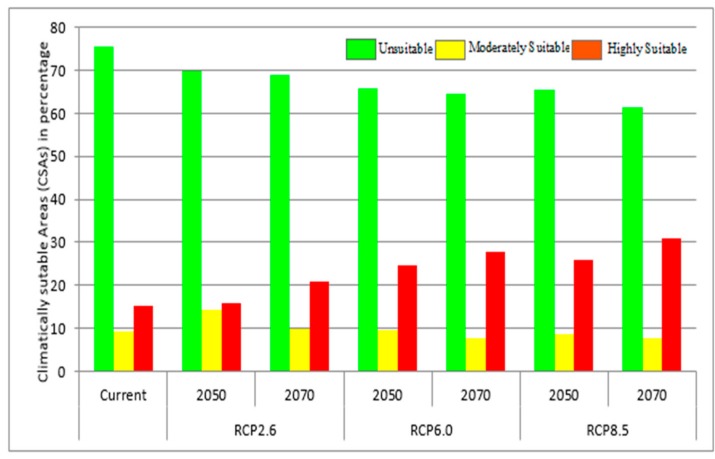
The predicted distribution of areas in Nepal climatically suitable for dengue fever at present and in the future. Green, yellow and red bars represent unsuitalbe, moderately suitable and highly suitable area, respectively.

**Figure 6:**
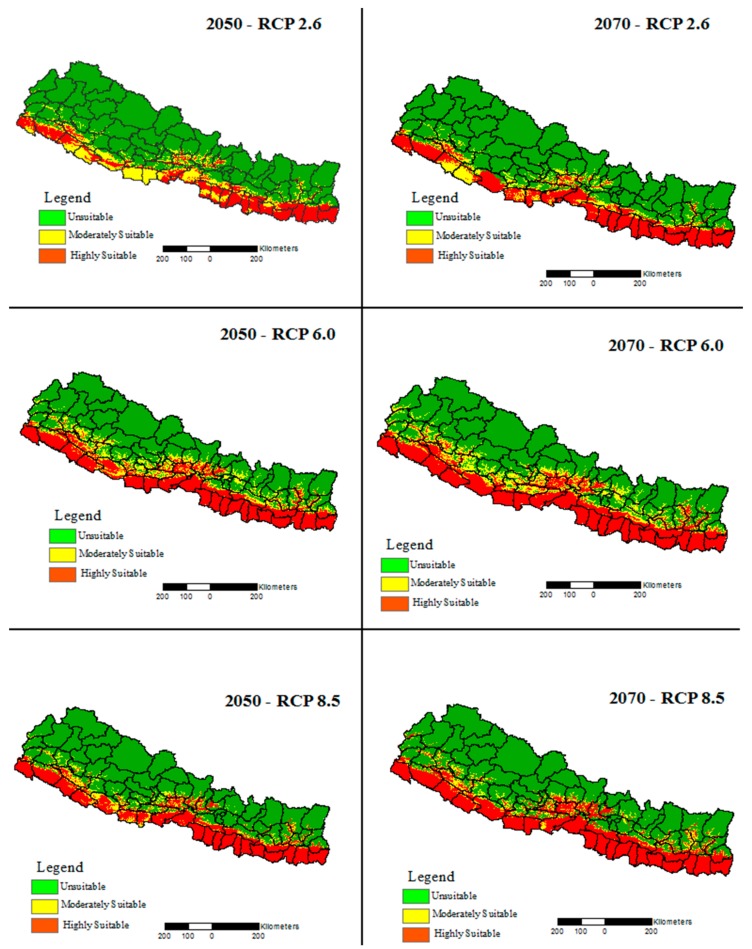
Spatial distribution of future climatically suitable areas of dengue fever in Nepal in 2050s and 2070s under the various greenhouse gas emission scenarios.

The estimated area of the predicted distribution is given in Figure 5 and that for the year of 2050 and 2070 under the different emission scenarios is presented in Figure 6. The climatically suitable area of dengue fever would increase in the 2050s and the 2070s under all the four RCPs, however the magnitude of the increase will vary based on time and emission scenario. The total climatically suitable areas would increase by larger amounts under the very high emission scenarios (RCP8.5) compared to the medium stabilization (RCP6.0) and mitigation emission scenario (RCP2.6). The proportions of moderately suitable areas would increase, and highly suitable areas remain constant in 2050 under the RCP2.6. However, the proportion of highly suitable areas would increase, and moderately suitable areas remain constant in 2070. But, in the other two emission scenario, the proportion of highly suitable areas would increase while the proportion of moderately suitable areas remain almost constant.

Spatial distribution of climatically suitable areas along the elevation gradient (Table 2) would be altered under all the future climate change emission scenarios compared to the present distribution (Figure 6).

Unlike the uniform rapid decline with increasing altitude in the present distribution, the climactically suitable areas would increasingly remain constant from 400–800 m asl in most of the RCPs (Figure S4). The maximum shift was observed for the 2070s under the very high emission scenarios (RCP8.5) where the upper altitudinal limit of climate suitable areas would reach up to 1400 m asl compared to that of below 900 m asl under the present distribution. The mean altitude of the climate niche of dengue would be shifted up to 464 m asl in the worst climate change emission scenarios compared to the 239 m asl of the present mean elevation.

**Figure 7:**
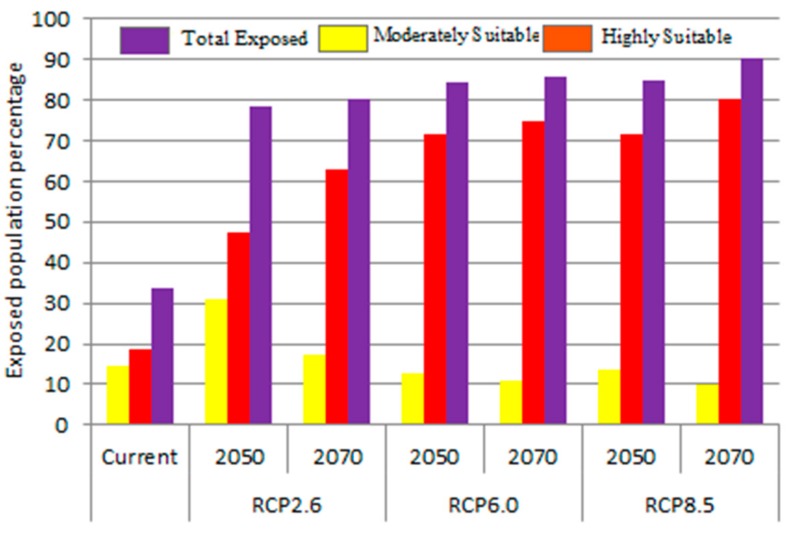
Proportion of human population under the risk of dengue fever in the present and future. The yellow bar reprsents the human population exposed to a moderately suitable area, the red bar to the highly suitable area and the magenta bar represents the total human poulation.

Our results also showed that 70.7% (14.7% in moderately suitable areas and 56% in highly suitable) of the human population of Nepal is presently exposed to the dengue fever climate niche when gridded population data was overlaid with the present MaxEnt model (Figure 7). The proportion of the exposed population would further increase in the all the RCPs compared to the presently exposed population with little variation among the years and the emission scenario assumed. For example, about 80% of the population would live in climatically suitable areas in the 2050s under mitigation emission scenario (RCP2.6). It would go above 90% under the very high emission scenarios (RCP8.5).

## 4. Discussion

In this study, we mapped present and future climatically suitable areas of dengue fever in Nepal using the reported dengue cases and a set of bioclimatic variables based on MaxEnt ecological niche modeling approach. Then, we estimated the present and future human population at risk of the disease based on the climate suitability model. In addition, we assessed the climatic niche shift of dengue fever along the elevation gradients by overlaying our present and future climatically suitable areas model with SRTM DEM.

Results of our model showed that temperature, especially the mean temperature of the wettest quarter (bio8) followed by the mean temperature of the coldest quarter (bio11) are major constraints on the distribution of the climatic niche of dengue fever in Nepal. These two variables accounted for almost 90% of the model. We observed nonlinear responses of climate variables which were consistent with previous research [15]. According to the response curve of our model, the mean temperature of the wettest quarter should be 25–30 °C and the mean temperature of the coldest quarter should be 14–18 °C to be climatically suitable areas. These temperature ranges reflect the thermal conditions necessary for the breeding and development of mosquito vectors during the breeding season and protect their eggs and larvae during the winter season. These ranges are concurrent with several previous studies [41,42].

Results of this study showed that the present predicted climactically suitable areas effectively captured the observed distribution of dengue fever in Nepal. Our model mapped all the 23 Tarai districts of Nepal as climatically suitable area for the dengue fever. The hot and humid southern lowland is suitable not only for dengue fever but also for other mosquito-borne diseases such as chikungunya, malaria, and encephalitis [26,43]. Most of the dengue fever cases and large outbreaks so far reported since its emergence were from these lowland Tarai [44]. A total of 917 laboratory confirmed dengue cases and five deaths were reported from lowland district Chitwan during the 2010 outbreak accounting 71% of total cases reported from the entire country. Three lowland Tarai districts—Chitwan, Jhapa, and Parsa—were badly affected by another large dengue outbreak in 2013 covering about 83% of total reported cases of the entire country. The moderately suitable area is generally distributed in the northern margins of the highly suitable areas. These areas coincide with the region of sporadic reporting of dengue cases. However, our model is slightly conservative in the west, especially for the Rupendehi, Kailali and Kanchanpur districts where the area mapped as moderately suitable was also expected to be the same as the highly suitable areas because of the prevailing hot and humid climate. Additionally, our model did not predict Kathmandu valley as climatically suitable at present despite continuous reporting of dengue fever [23,45]. Dengue cases and the vector of the disease has been reported at even higher altitudes (1800 m asl) [46,47] in recent years. This showed little mismatch on our model along the edge. The uncertainty analysis of the previous ecological niche model showed higher incongruence generally along the edge [48] compared to the core areas. Such incongruent results along the edge of the bioclimatic niche of dengue was also observed in Tanzania [49], like our study. However, the dengue cases reported from Kathmandu valley could be imported cases from lowland Tarai and neighboring districts such as Dhading and Nuwakot; that might be due to frequent travel of people to the country capital where administrative and medical services are centralized. Nonetheless, our model validation suggests (high AUC) a high level of accuracy of the model.

Like several previous studies [1,14,18,49], our study also suggested a subtle shift in the present climatically suitable areas for both the 2050s and 2070s. According to our model, the upper limit of the climate niche of dengue fever will reach up to 1400 m asl compared to the present-day limit of 900 m asl. Kathmandu valley will be climatically suitable for dengue fever in both the 2050s and 2070s giving further threats to the highly-populated capital city. The magnitude of the change, however, will vary according to the emission scenarios assumed. For example, it will be at a minimum under the RCP2.6 and at a maximum under the RCP8.5. The projected areas of range expansion are primarily expected along the edge and proximate to the present distribution [50]. The altitudinal shift of climatically suitable areas of dengue fever is generally consistent with other climate modeling which report species movement to higher latitudes and elevations in response to warming [51]. Moderately suitable areas changing to highly suitable areas indicates the possibility of large and frequent dengue outbreaks in these areas in the future. Circulating all four dengue virus serotypes among the vector, host, and environment also increases the risk of sequential infection with different dengue serotypes and dengue hemorrhagic fever [28,29]. Our estimates suggest that the population living at risk of dengue fever is likely to increase significantly in the future, which is consistent with studies made earlier at a global scale [14]. In the worst emission scenarios (RCP8.5), the population at risk of dengue is expected to be about 90% in 2070s which is fairly large compared to the worldwide predicted value of 52% in the 2085. This indicates more resources will be needed to tackle dengue fever in the future, which could be a great challenge to the public health authority in Nepal.

Our study defined for the first time the present and future spatial limit of the climatic niche of dengue fever in Nepal. Therefore, this study provides crucial baseline information necessary to design surveillance and allocate scarce resources effectively. However, there were several limitations associated with this study which should be taken into consideration while interpreting our results. For instance, the availability of dengue case data in Nepal is limited. Therefore, we used newspaper portals and the dengue health map as alternative sources of dengue case data. Hence, the accuracy of our model depends on the accuracy of reports. Further, our model only showed climatically suitable areas. Although a suitable climate is necessary for dengue transmission, several other non-climate socioeconomic and proximate factors are needed for an epidemic to take place. The importance of such non-climate factors increases with increasing the resolution of the study [52]. The results of the modelling strongly depend on the choice of input variables [53] and modelling approach adopted [34]. Therefore, mapping results may not match when the model is calibrated based on other climatic variables. Similarly, like other spatial models, our study cannot interpret the temporal risk window for particular areas, for example, whether the climatically suitable areas have a risk of dengue the whole the year round or in just some of months of the year. Several studies have shown that the temporal window of the risk would be widening due to climate change [18,54]. Depending of the climate, some areas could be prone for short periods while other areas for a little longer. Future studies should focus on the temporal risk period along with the spatial risk. Similarly, we assumed continuous population growth which, in reality, might not be true.

## 5. Conclusions

In this study, we mapped present and future areas climactically suitable for dengue fever in Nepal for the first time based on the MaxEnt ecological niche modelling approach using the reported dengue cases and a set of bioclimatic variables. Our results showed that about one quarter of the area of Nepal, mainly in the southern lowland Tarai, is currently suitable for dengue fever. This will expand northwards in the future in response to climate change. Our estimate suggests that 70% of the total population is currently at risk of dengue fever and that would further increase (by up to 90%) in the future due to climate change. The results along with the map produced in this study could guide health authorities to implement effective surveillance and control strategies of dengue fever in Nepal.

## Figures and Tables

**Table 1:** Predictor variables used in the construction of the niche models.

Abbreviation	Description
Bio1	Annual Mean Temperature
Bio2	Mean Diurnal Range (Mean of monthly (max temp–min temp))
Bio3	Isothermally (P2/P7) (×100)
Bio4	Temperature Seasonality (standard deviation × 100)
Bio5	Max Temperature of Warmest Month
Bio6	Min Temperature of Coldest Month
Bio7	Temperature Annual Range (P5–P6)
Bio8	Mean Temperature of Wettest Quarter
Bio9	Mean Temperature of Driest Quarter
Bio10	Mean Temperature of Warmest Quarter
Bio11	Mean Temperature of Coldest Quarter
Bio12	Annual Precipitation
Bio13	Precipitation of Wettest Month
Bio14	Precipitation of Driest Month
Bio15	Precipitation Seasonality (Coefficient of Variation)
Bio16	Precipitation of Wettest Quarter
Bio17	Precipitation of Driest Quarter
Bio18	Precipitation of Warmest Quarter
Bio19	Precipitation of Coldest Quarter

**Table 2:** Distribution of climate suitable areas along the elevation gradient.

Climate Trajectories	Year	Min (m asl)	Mean (m asl)	Max (m asl)
	Present	61	239	894
RCP2.6	2050	61	338	1060
RCP2.6	2070	61	324	1167
RCP6.0	2050	61	390	1183
RCP6.0	2070	61	411	1297
RCP8.5	2050	61	398	1249
RCP8.5	2070	61	464	1388

## Supplementary Materials

The following are available online at http://www.mdpi.com/1660-4601/15/2/187/s1, Note S1. R code used for geocoding address level dengue occurrence point using Google API; Table S1: Dengue presence locations; Table S2: Correlation matrix of bioclimatic variables; Figure S1: Jackknife of regularized training gain; Figure S2: Response curves for climate variables related to the distribution of dengue fever; Figure S3: Distribution of Present Climate suitable pixel along the elevation gradient; Figure S4: Distribution of Future Climate suitable pixel in different concentration pathways along the elevation gradient.
