# Supplementary material for: Present and Future of Dengue Fever in Nepal: Mapping Climatic Suitability by Ecological Niche Model
Source: Int J Environ Res Public Health. 2018 Jan 23;15(2):187. doi: 10.3390/ijerph15020187 (PMC5857046; doi:10.3390/ijerph15020187)
Supplement: Supplementary file 1 [file ijerph-15-00187-s001.pdf]

Supplementary Note S1. R code used for geocoding address level dengue occurrence point using Google API,

```
#install.packages('RCurl')
#install.packages('RJSONIO')
#install.packages('plyr')

library(RCurl)
library(RJSONIO)
library(plyr)
url <- function(address, return.call = "json", sensor = "false") {
 root <- "http://ditu.google.cn/maps/api/geocode/"
 u <- paste(root, return.call, "?address=", address, "&sensor=", sensor, sep = "")
 return(URLencode(u))
}
geoCode <- function(address,verbose=FALSE) {
 if(verbose) cat(address,"\n")
 u <- url(address)
 doc <- getURL(u)
 x <- fromJSON(doc,simplify = FALSE)
 if(x$status=="OK") {
 lat <- x$results[[1]]$geometry$location$lat
 lng <- x$results[[1]]$geometry$location$lng
 location_type <- x$results[[1]]$geometry$location_type
 formatted_address <- x$results[[1]]$formatted_address
 return(c(lat, lng, location_type, formatted_address))
 } else {
 return(c(NA,NA,NA, NA))
 }
}

address <- geoCode("Birta, Bara, Nepal")
```

Supplementary Table S2: Spatially filtered 124 Dengue presence locations.

S.N.	x	y
1	80.16558477900	28.98705699980
2	80.32141811230	28.92955699980
3	80.32141811230	28.83705699980
4	80.89891811230	28.83122366650
5	80.61058477890	28.78872366650
6	80.48975144560	28.77039033320

7	80.56975144560	28.74039033320
8	80.57475144560	28.71039033320
9	81.06475144560	28.69205699980
10	80.43975144560	28.68039033320
11	81.62558477890	28.57789033320
12	81.32641811220	28.45372366650
13	81.52891811220	28.25622366650
14	83.93891811210	28.25289033320
15	83.97891811210	28.25289033320
16	81.44891811220	28.24789033320
17	83.93891811210	28.24289033320
18	82.29141811220	28.12205699990
19	81.61891811220	28.06539033320
20	81.61475144550	28.05455699990
21	82.47391811220	28.02622366660
22	82.50475144550	27.80789033320
23	84.49058477870	27.78039033320
24	84.54975144530	27.77039033320
25	84.45225144540	27.73372366660
26	84.36475144540	27.72872366660
27	84.19641811200	27.72122366660
28	85.31975144530	27.72122366660
29	85.31641811200	27.71705699990
30	85.32391811200	27.71705699990
31	84.21225144540	27.71622366660
32	83.44975144540	27.70039033320
33	84.43058477870	27.69872366660
34	84.42641811200	27.69455699990
35	84.43141811200	27.69455699990
36	85.41558477860	27.69289033320
37	84.43058477870	27.68122366660
38	84.18058477870	27.67955699990
39	84.41891811200	27.67622366660
40	83.45558477880	27.67539033330
41	83.99975144540	27.66705699990
42	84.53808477870	27.64789033330
43	84.61641811200	27.64455699990
44	84.16308477870	27.63705699990
45	84.50641811200	27.62789033330
46	83.64558477870	27.62705699990
47	84.51141811200	27.61705699990

48	83.95225144540	27.61622366660
49	84.56558477870	27.61622366660
50	84.64308477870	27.60455699990
51	84.36475144540	27.58789033330
52	84.49975144540	27.58372366660
53	84.78391811200	27.55789033330
54	83.04725144540	27.55205699990
55	84.35391811200	27.52872366660
56	83.46058477880	27.51039033330
57	82.79391811210	27.50955699990
58	83.41641811210	27.50539033330
59	83.46975144540	27.47789033330
60	83.77808477870	27.47539033330
61	83.90558477870	27.45789033330
62	84.34391811200	27.44705699990
63	85.00225144530	27.43705699990
64	85.02808477860	27.43205699990
65	85.02891811200	27.42289033330
66	84.42808477870	27.40372366660
67	84.89225144530	27.34372366660
68	85.12725144530	27.20705699990
69	84.70558477870	27.19705699990
70	84.99225144530	27.19039033330
71	84.91391811200	27.16955700000
72	84.78641811200	27.10372366660
73	85.57141811190	27.09205700000
74	84.91558477870	27.06872366660
75	85.34558477860	27.06289033330
76	84.76058477870	27.05539033330
77	85.32391811200	27.04539033330
78	84.88058477870	27.04205700000
79	84.75475144530	27.03789033330
80	84.91558477870	27.03372366660
81	85.00808477860	27.03205700000
82	84.88558477870	27.03039033330
83	84.94891811200	27.03039033330
84	84.88641811200	27.02372366660
85	84.83308477870	27.02289033330
86	84.88975144530	27.02289033330
87	84.87725144530	27.01872366660
88	84.88641811200	27.01872366660

89	84.88891811200	27.01872366660
90	84.87725144530	27.01705700000
91	84.88391811200	27.01455700000
92	84.88058477870	27.01289033330
93	84.88475144530	27.01289033330
94	84.87225144530	27.01122366660
95	84.87308477870	27.00539033330
96	84.86641811200	27.00039033330
97	84.86641811200	26.99872366660
98	85.27725144530	26.95955700000
99	88.15308477840	26.75289033330
100	88.08058477840	26.74872366660
101	88.03975144510	26.72372366660
102	88.04891811180	26.72289033330
103	88.17141811180	26.72039033330
104	88.01391811180	26.69622366660
105	88.03391811180	26.68455700000
106	87.70058477850	26.67289033330
107	87.66808477850	26.67205700000
108	88.09475144510	26.67205700000
109	88.15891811180	26.66955700000
110	87.69475144510	26.66622366670
111	88.12058477840	26.66205700000
112	88.13891811180	26.65539033330
113	88.15558477840	26.64622366670
114	87.98391811180	26.64539033330
115	88.02725144510	26.64122366670
116	87.99391811180	26.63372366670
117	87.98225144510	26.62955700000
118	88.10141811180	26.54122366670
119	86.84475144520	26.50539033330
120	87.28391811180	26.48039033330
121	87.27641811180	26.47289033330
122	87.29391811180	26.46205700000
123	87.27141811180	26.45289033330
124	87.26891811180	26.42455700000

Table S3: Correlation matrix of bioclimatic variables

Code	Bio1	Bio2	Bio3	Bio4	Bio5	Bio6	Bio7	Bio8	Bio9	Bio10	Bio11	Bio12	Bio13	Bio14	Bio15	Bio16	Bio17	Bio18	Bio19
Bio1	1	0.26908	-0.64211	0.78213	0.27301	0.36429	0.06239	-0.03409	-0.00526	-0.00457	-0.58729	0.14839	-0.05505	-0.52015	0.08308	-0.6264	0.94857	0.3002	0.02297
Bio2		1	0.03292	0.30453	0.99326	0.98177	0.71097	0.62659	0.58967	0.67785	-0.46961	0.73497	0.56799	-0.58497	-0.07708	-0.60626	0.12834	0.79096	0.68069
Bio3			1	-0.51885	0.06626	-0.12845	0.27039	0.40958	0.36625	0.36019	0.77752	0.1882	0.45486	0.67639	-0.51148	0.61045	-0.60046	-0.29261	0.32915
Bio4				1	0.30665	0.39653	0.01996	-0.07026	-0.04191	-0.03955	-0.57503	0.09532	-0.10353	-0.48848	0.22354	-0.54678	0.70113	0.37858	-0.01841
Bio5					1	0.96795	0.70495	0.62855	0.5932	0.67612	-0.43248	0.72774	0.57361	-0.54169	-0.08954	-0.57059	0.14221	0.77462	0.67809
Bio6						1	0.63279	0.52324	0.48429	0.58697	-0.60723	0.66838	0.45444	-0.69852	0.04807	-0.69331	0.20565	0.86215	0.59215
Bio7							1	0.98323	0.9577	0.99243	-0.07179	0.99466	0.96298	-0.29839	-0.58454	-0.42698	0.00119	0.24577	0.99716
Bio8								1	0.97644	0.99382	0.10788	0.96457	0.99502	-0.11994	-0.68048	-0.27214	-0.06524	0.11622	0.99385
Bio9									1	0.95777	0.10958	0.94553	0.9767	-0.11406	-0.71085	-0.29264	0.00327	0.06513	0.96682
Bio10										1	0.02657	0.97744	0.97978	-0.19769	-0.61664	-0.32935	-0.06151	0.20115	0.99786
Bio11											1	-0.15321	0.1925	0.95677	-0.50774	0.87808	-0.42971	-0.72254	0.00062
Bio12												1	0.94022	-0.36881	-0.55738	-0.50644	0.08332	0.28493	0.98609
Bio13													1	-0.03649	-0.72848	-0.20028	-0.06536	0.03393	0.97942
Bio14														1	-0.36533	0.91147	-0.35825	-0.71473	-0.22654
Bio15															1	-0.04042	-0.00997	0.44569	-0.62283
Bio16																1	-0.50064	-0.59965	-0.36401
Bio17																	1	0.11982	-0.02858
Bio18																		1	0.19754
Bio19																			1

Supplementary Figure S4: a) Jackknife of regularized training gain for individual bioclimatic variables and b) Jackknife of regularized training gain omitting each bioclimatic variable

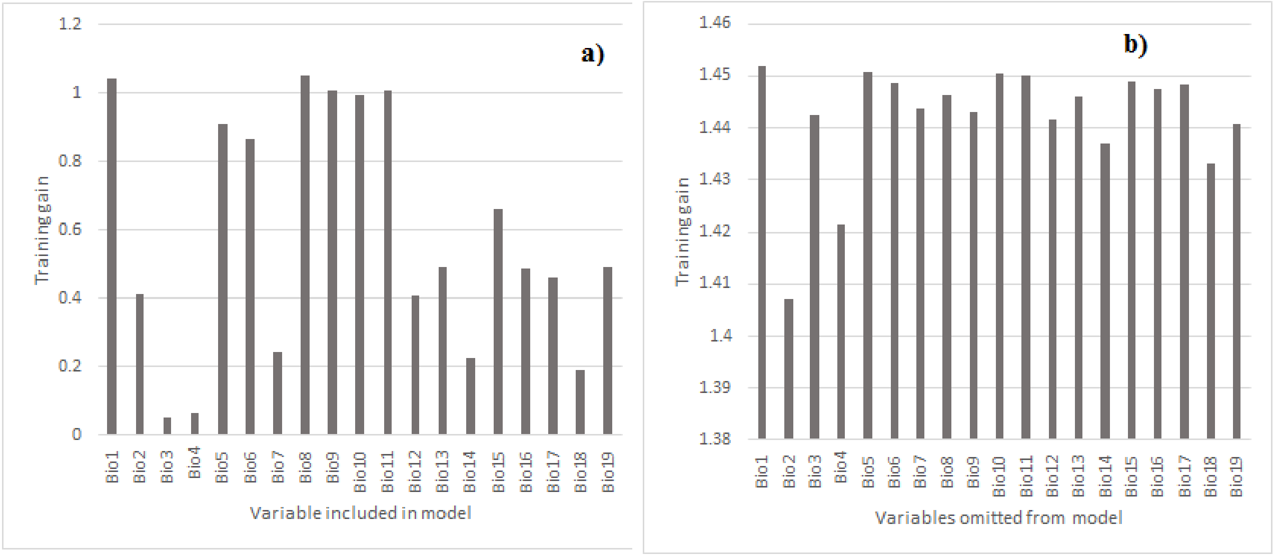

Supplementary Figure S5: Response curves for climate variables related to the distribution of dengue fever. The red line is the mean value of 30 Maxent runs and blue bar represents +_1 SD

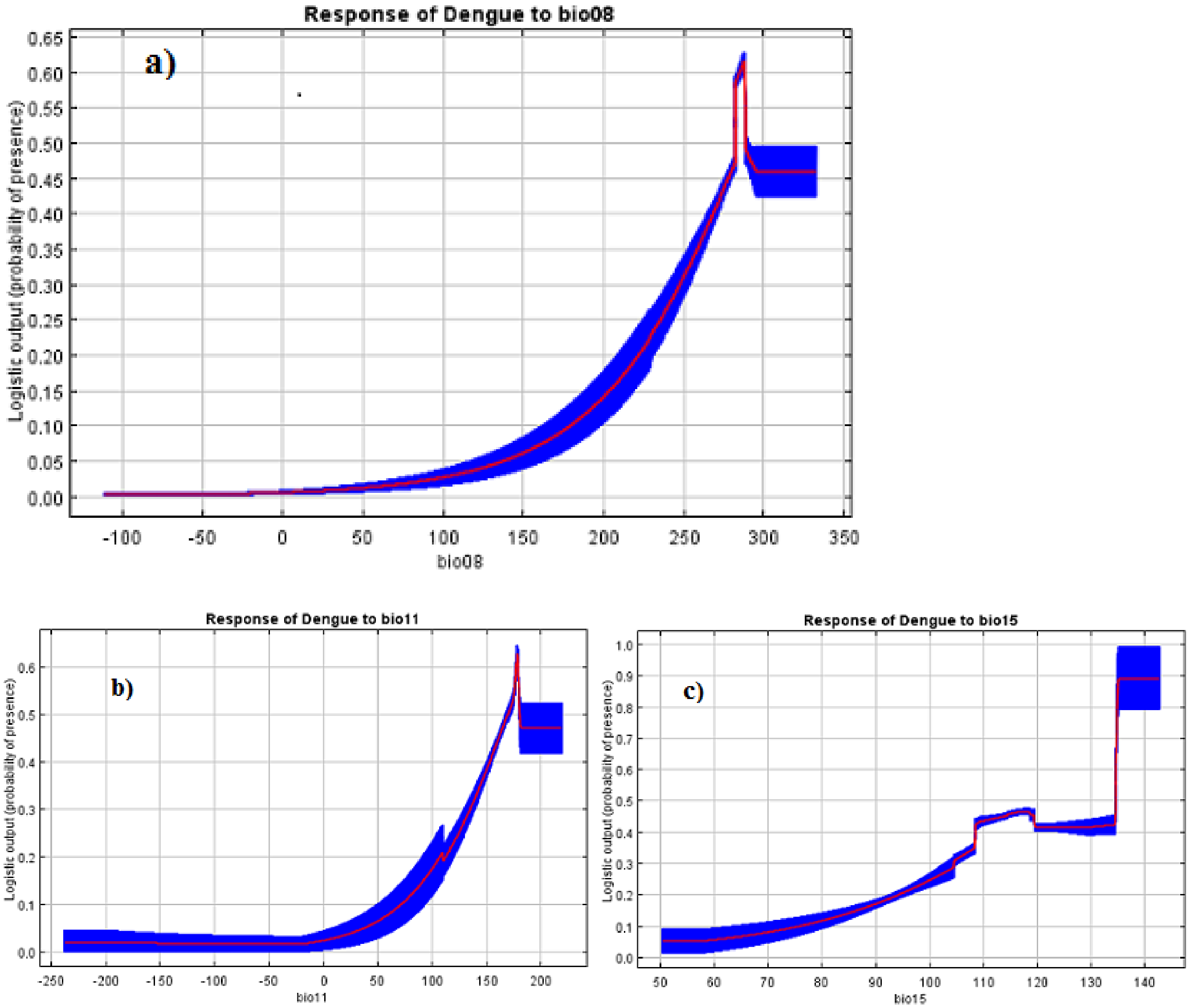

Supplementary Figure S6: Distribution of Present Climate suitable pixel along the elevation gradient. The x axis represent elevation gradient and y axis represents the pixel frequency. Yellow bar reprsents pixel frequency in moderately suitable area, red bar to the highly suitable area and magenta repsents total pixel.

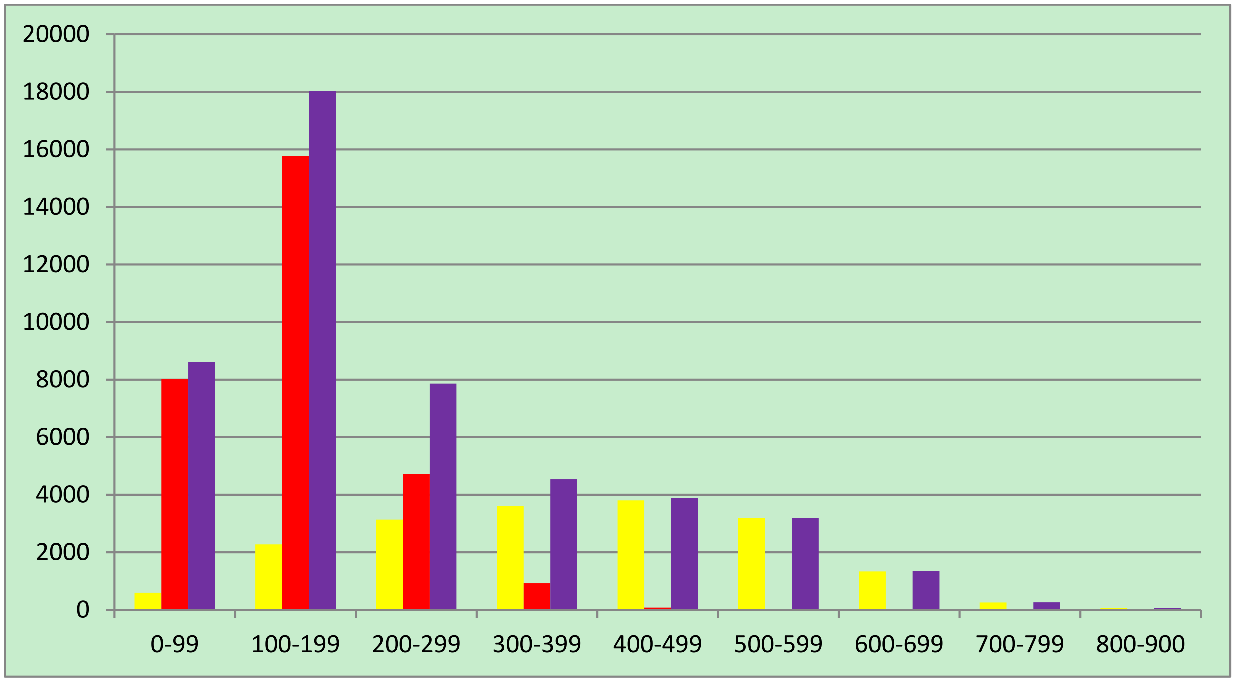

Supplementary Figure S7: Distribution of Future Climate suitable pixel in different along the elevation gradient. The x axis represent elevation gradient and y axis represents the pixel frequency. Yellow bar reprsents pixel frequency in moderately suitable area, red bar to the highly suitable area and magenta repsents total pixel.

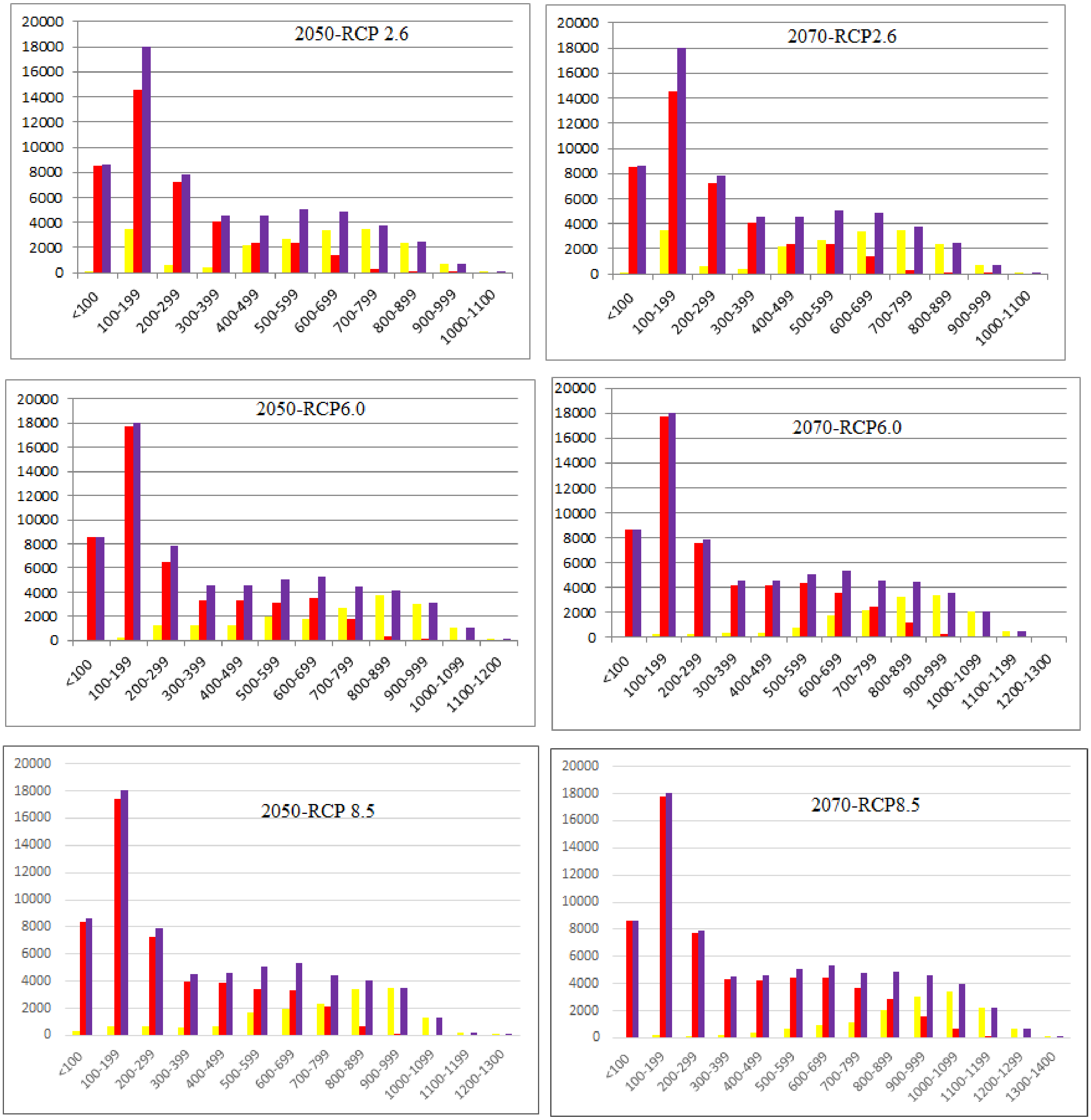
2050-RCP 2.6
2070-RCP2.6
2050-RCP6.0
2070-RCP6.0
2050-RCP 8.5
2070-RCP8.5
20000
18000
16000
14000
12000
10000
8000
6000
4000
2000
0
<100
100-199
200-299
300-399
400-499
500-599
600-699
700-799
800-899
900-999
1000-1099
1100-1199
1200-1299
1300-1400
1000-1100
1100-1200
1200-1300
